# Novel Mechanical Aspiration Thrombectomy in Patients With Acute Pulmonary Embolism: Results From the Prospective APEX-AV Trial

**DOI:** 10.1016/j.jscai.2024.102463

**Published:** 2024-12-27

**Authors:** Mona Ranade, Malcolm T. Foster, Paul S. Brady, Seth I. Sokol, Sabah Butty, Andrew Klein, Robert Maholic, Ammar Safar, Taral Patel, David Zlotnick, Daniel Gans, Jeffrey Pollak, Dean Ferrera, Brian Stegman, Sukhdeep Basra, John Moriarty, Brent Keeling

**Affiliations:** a Division of Interventional Radiology, Department of Radiology, David Geffen School of Medicine at UCLA, Los Angeles, California; b Interventional Cardiology, Tennova Healthcare Turkey Creek Medical Center, Knoxville, Tennessee; c Section of Interventional Radiology, Department of Radiology, Einstein Health Care Network, Philadelphia, Pennsylvania; d Division of Cardiology, Department of Medicine, Jacobi Medical Center and the Albert Einstein College of Medicine, Bronx, New York; e Department of Radiology, Indiana University School of Medicine, Indianapolis, Indiana; f Interventional Cardiology, Vascular and Endovascular Medicine, Piedmont Heart Institute, Atlanta, Georgia; g Interventional Cardiology, University of Pittsburgh Medical Center Hamot, Erie, Pennsylvania; h Interventional Cardiology, Kettering Health, Miamisburg, Ohio; i HonorHealth Research Institute, HonorHealth, Scottsdale, Arizona; j Division of Cardiovascular Medicine, Gates Vascular Institute, Buffalo General Medical Center, University at Buffalo, Buffalo, New York; k Interventional Radiology, OSF HealthCare, Peoria, Illinois; l Vascular and Interventional Radiology, Yale School of Medicine, New Haven, Connecticut; m Department of Cardiology, Community Hospital, Munster, Indiana; n Interventional Cardiology, CentraCare Heart and Vascular Center, St. Cloud, Minnesota; o Center for Advanced Heart Failure, University of Texas Health Science Center and Memorial Hermann Hospital, Houston, Texas; p Division of Cardiothoracic Surgery, Emory University School of Medicine, Atlanta, Georgia

**Keywords:** AlphaVac, aspiration thrombectomy, intermediate-risk pulmonary embolism, mechanical thrombectomy, thrombus, vacuum aspiration

## Abstract

**Background:**

There is a need for additional data to assess procedural efficacy and risks associated with mechanical thrombectomy for treating pulmonary embolism (PE) due to its increased utilization and diversity of patient populations presenting with PE. This study evaluated the safety and efficacy of percutaneous mechanical aspiration thrombectomy with the AlphaVac F18^85^ System (AngioDynamics) in patients with acute intermediate-risk PE.

**Methods:**

Patients with acute intermediate-risk PE and a right ventricular (RV)/left ventricular (LV) diameter ratio of ≥0.9 were eligible for enrollment in this prospective, multicenter, single-arm study. The primary effectiveness end point was reduction in the RV/LV ratio at 48 hours. The primary safety end point was the rate of major adverse events (MAEs) defined as subjects who experienced major bleeding, device-related deaths, clinical deterioration, or pulmonary vascular or cardiac injury within 48 hours postprocedurally.

**Results:**

In total, 122 subjects were enrolled at 25 sites. Mean procedure time was 37.2 ± 17.7 minutes. There were statistically significant reductions in mean 48-hour postprocedural RV/LV diameter ratio (−0.45 ± 0.27; *P* < .001). Postprocedural mean pulmonary arterial pressure also significantly declined from 27.8 ± 7.8 mm Hg before the procedure to 21.8 ± 7.2 mm Hg (*P* < .001). There was a 35.5% mean reduction in clot burden as measured by the modified Miller index score. Five (4.1%) subjects developed 7 MAEs during the postprocedural 48-hour assessment period, the majority of which were access site bleeding.

**Conclusions:**

Percutaneous mechanical aspiration thrombectomy with the AlphaVac system provided a safe and effective treatment for acute intermediate-risk PE with a significant reduction in RV/LV ratio and clot burden with a low rate of adverse events.

## Introduction

Pulmonary embolism (PE) represents the third leading cause of cardiovascular mortality in the United States.^1^ Patients with intermediate-risk PE account for 35% to 55% of hospitalized patients presenting with PE and have an associated mortality rate of 5% to 24%.^1^^,^^2^ For such patients, therapies aim to prevent possible hemodynamic collapse and death resulting from progressive right-sided heart failure and to expedite symptom resolution.^1^

The current standard of care for treatment of PE is therapeutic anticoagulation. Specific to patients with intermediate-risk PE, the ACC/AHA guidelines discourage routine administration of thrombolytic therapy (either systemic or catheter directed) and recommends that patients should be promptly anticoagulated, receive supportive measures, and be closely monitored.^1^ Adverse outcomes despite anticoagulation in patients with intermediate-risk PE, however, have prompted treating physicians to consider therapeutic escalation through systemic thrombolysis, catheter-directed therapies, or surgical embolectomy.^1^ Catheter-based approaches to treatment are options for patients who remain hemodynamically stable with an elevated risk of decompensation or bleeding risk.

While mechanical aspiration thrombectomy has been shown to be effective in the treatment of PE, its use has been shown to be associated with device-related complications, difficulties in cannula navigation, and the presence of postthrombectomy residual emboli.^3^ To overcome some of these limitations, a percutaneous mechanical aspiration thrombectomy device (AlphaVac F18^85^ system; AngioDynamics) was developed to improve procedural safety efficiency and to ease device navigation. In this study, we report the results from the prospective Investigational Device Exemption (IDE) trial, APEX-AV (Acute Pulmonary Embolism extraction with the AlphaVac system) designed to evaluate the safety and effectiveness of AlphaVac F18^85^ system in patients with acute intermediate-risk PE.

## Materials and methods

### Study design

The APEX-AV study was a prospective, single-arm, multicenter study in which patients with acute intermediate-risk PE underwent treatment with a percutaneous mechanical aspiration thrombectomy device. The study was conducted in partnership with the Pulmonary Embolism Response Team (PERT) Consortium. The study was registered at ClinicalTrials.gov (NCT05318092) and was approved by a central ethics committee (WCG Clinical) and several institutional ethics committees. All subjects who participated in the study signed written informed consents.

### Study population

Patients aged 18 y or older presenting with clinical signs and symptoms consistent with acute intermediate-risk PE for 14 days or less and confirmed by computed tomography angiography (CTA) were eligible for enrollment in the study. Subjects were also required to have a right ventricular (RV)/left ventricular (LV) diameter ratio ≥0.9 noted on CTA, systolic blood pressure ≥90 mm Hg, and a heart rate ≤130 beats per minute before the procedure. Exclusion criteria included subjects with any contraindication to systemic or therapeutic doses of heparin or anticoagulants, thrombolytics (tissue-plasminogen activator) therapy within the past 30 days before CTA, or pulmonary hypertension with peak systolic pulmonary artery pressure (PAP) >70 mm Hg. The full list of inclusion and exclusion criteria are reported in Supplemental Table S1.

### Device description

**Figure 1:**
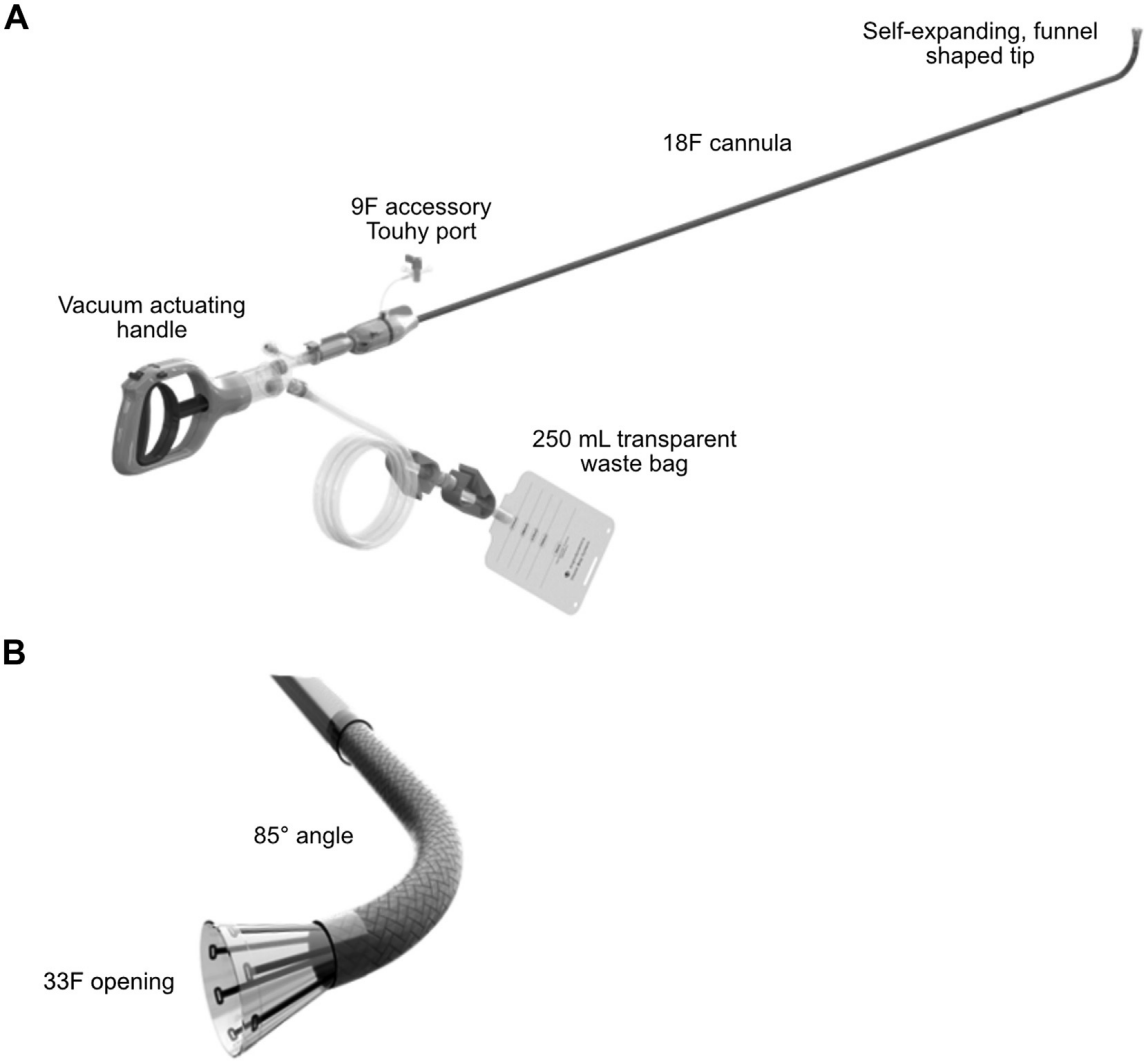
**The AlphaVac system.** (A) Handle, cannula, and waste bag, and (B) 85 degree angled cannula tip APEX-AV, Acute Pulmonary Embolism extraction with the AlphaVac system.

The AlphaVac system consists of an ergonomic handle that acts as the vacuum source, a large 18F cannula with a funnel tip that expands to 33F, and a waste bag that can hold up to 250 mL of blood and clot material (Figure 1). The handle also has a volume-limiting switch, which can be set to either 10 or 30 mL per pull to minimize blood loss. The device generates suction pressure close to perfect vacuum with each 30-mL pull of the device handle when the funnel is fully occluded. The device also has a vacuum lock mechanism that maintains this negative pressure, permitting single-hand operation. The expandable funnel tip is designed to prevent clogging of the cannula when removing large amounts of material. The clear waste bag enables the operator to visually monitor and measure blood loss and aspirated material removed during the procedure.

### Procedural details

CTA was obtained for all subjects before the procedure. All subjects were heparinized during the procedure and either the right internal jugular vein or femoral vein was accessed and used for the placement of a 22F sheath. Preparing the device for insertion included assembly and priming of the F18 sheath and obturator, and the AlphaVac handle, cannula, waste line, and bag. Following assembly and priming steps for the F18 sheath and obturator, it was advanced over a stiff wire into the desired location within the pulmonary vasculature, and the wire and dilator were removed. The 18F cannula was inserted into the sheath and advanced to the first treatment zone within the pulmonary artery (PA). The intended direction of treatment was from proximal to distal, and the device was designed to navigate through, and extract thrombus from, the pulmonary vasculature without a wire (although the ability to work over-the-wire is an option). Once the cannula was delivered to the treatment zone, a volume-limiting switch on the device handle was then set to either 10 or 30 mL. With an 85° angled tip, operators have the ability to navigate the cannula from one PA to the other without using a guide wire. Once the emboli were engaged, the aspiration handle was pulled back pulling the material into the cannula, removing it from the vasculature. Once the thrombus was aspirated from each of the target locations, the device and the 22F access sheath were removed, and the venous entry site was closed. Hemostasis was achieved with either a simple skin closure and light pressure was held or a venous closure device was used to close the venotomy.

### Effectiveness end points

The primary effectiveness end point was the reduction in the RV/LV ratio at 48 hours postprocedurally assessed by CTA. The primary effectiveness was assessed as the difference between the baseline and the 48-hour RV/LV diameter ratio. An independent Core Lab reviewed both the preprocedural and postprocedural imaging scans to determine the RV/LV diameters.

Additional effectiveness end points included intensive care unit (ICU) and hospital length of stay, the use of thrombolytics during and within 48 hours of the procedure and the change in modified Miller index score and mean PAP from baseline following the procedure. While the method for measuring preprocedural and postprocedural PAP was not standardized and left to the discretion of the operator, all measurements were performed using right heart catheterization or echocardiography.

### Safety end points

The primary safety end point was the rate of major adverse events (MAEs) postprocedurally defined as the number of subjects developing Global Utilization of Streptokinase and Tissue Plasminogen Activator for Occluded Coronary Arteries (GUSTO) defined major bleeding, device-related deaths, or device-related clinical deterioration, pulmonary vascular injury, or cardiac injury within 48 hours of the procedure. Supplemental Table S2 lists the definitions for major bleeding, clinical deterioration, pulmonary vascular injury, and cardiac injury. Bleeding was classified by the GUSTO bleeding criteria with major bleeding defined as bleeding included in the GUSTO severe or moderate categories. Severe bleeding included the presence of intracranial hemorrhage, fatal or life-threatening bleeding, or bleeding resulting in substantial hemodynamic compromise requiring intervention. Moderate bleeding was defined as bleeding that resulted in the need for blood transfusion but was not associated with hemodynamic compromise. Secondary safety end points also included the rate of serious and nonserious device-related adverse events or symptomatic recurrence of PE within 30 days of the procedure date.

### Statistical analysis

The effectiveness goal was a 0.12-mm reduction in the RV/LV ratio from baseline was based on the mean decrease in RV/LV diameter ratio across the 4 randomized controlled trials reporting results associated with the treatment of acute PE with anticoagulants.^4^, ^5^, ^6^, ^7^ An SD of 0.25 mm for the effectiveness goal was based on the SD of the RV/LV diameter ratio observed in a prospective study of a percutaneous mechanical thrombectomy device in patients with acute intermediate-risk PE.^8^

The safety performance goal for the study was less than 25% of subjects experiencing an MAE. This was based on the upper limit of the 95% CI for the average composite MAE rate (16.3%) observed across 7 randomized controlled trials in which acute PE was treated with thrombolysis versus an anticoagulant control.^4^^,^^6^, ^7^, ^8^, ^9^, ^10^, ^11^, ^12^ The hypothesized composite MAE rate in this study was expected to be approximately 15%. To detect a difference between an expected MAE rate of 15% and the performance goal of 25% with 80% power, the necessary sample size was calculated to be 103 (with a 1-sided *P* value = .05). A sample size of 103 patients was selected as a conservative sample size to assess safety with adequate statistical power. To account for an attrition rate of 15%, 122 subjects were projected to be enrolled.

Results are reported for the as treated subject population defined as subjects who met the inclusion/exclusion criteria and in whom the cannula for the mechanical aspiration thrombectomy device was placed in the jugular or 1 of the common femoral veins. The statistical analyses conducted for quantitative variables included frequency counts and percentages, means, SD, minimum, and maximum. Categorical variables were summarized by frequencies and percentages. Unless explicitly stated, percentages used a denominator corresponding to the number of unique patients who contributed to the end point. For the primary effectiveness end point, mean reduction in the RV/LV ratio was compared with the performance goal of 0.12 using a paired-sample *t* test with a significance level of 0.05. For the primary safety end point, the rate of any MAE within 48 hours of the procedure was compared with the performance goal of 25% using a 1-sample proportion test with a significance level of .05. All statistical analyses were performed using SAS statistical software version 9.2 (SAS Institute).

## Results

Between October 2022 and December 2023, 122 subjects with acute intermediate-risk PE were enrolled at 25 US sites. A total of 118 subjects completed the study with 3 (2.5%) subjects lost to follow-up and 1 (0.8%) withdrawing from the study. Baseline subject demographics and characteristics are listed in Table 1. The mean age was 61.9 ± 14.6 years with 68 (55.7%) female subjects. The majority of the subjects (86.9%) presented with bilateral PE with or without central pulmonary emboli. One hundred and twenty (98.4%) subjects were on anticoagulation when enrolled in the study. A total of 111 (91.0%) subjects had elevated levels of troponin or natriuretic peptide.

**Table 1:** Patient demographics and clinical characteristics

Characteristic	Value
Age, y	61.9 ± 14.6
Sex	
Male	54 (44.3)
Female	68 (55.7)
Race	
Black or African American	34 (27.9)
White	84 (68.9)
Unknown	4 (3.3)
Ethnicity	
Hispanic or Latino	5 (4.1)
Not Hispanic or Latino	113 (92.6)
Unknown	4 (3.3)
Smoking status	
Current	15 (12.3)
Former	29 (23.8)
Never	78 (63.9)
Computed tomography angiography (RV/LV ratio)	1.52 ± 0.31
Modified Miller index	15.2 ± 1.62
Pulmonary artery pressure, mm Hg	27.8 ± 7.81
Clot location	
Bilateral + central	39 (32.0)
Bilateral only	67 (54.9)
Unilateral left	5 (4.1)
Unilateral right	10 (8.2)
Unknown	1 (0.8)

Values are mean ± SD or n (%).

### Periprocedural characteristics

Procedural and postoperative details are reported in Table 2. The femoral vein was used for all procedures with the right femoral vein used for 115 (94.3%) of the cases. The majority of procedures were performed under conscious sedation (82.8%), with a mean duration of 37.2 ± 17.7 minutes. Only 1 device malfunction occurred during the study. This consisted of a device not suctioning as intended during the procedure with the investigator discontinuing the use of the device and replacing it with a new one, which functioned properly. One subject received an intraoperative transfusion, and 3 (2.5%) subjects received thrombolytic infusions, all during the procedure at the discretion of the treating provider. The mean ICU and total hospital length of stay were 1.5 ± 1.8 and 5.3 ± 3.3 days, respectively.

**Table 2:** Perioperative details

Characteristics	Value
Access site	
Right femoral vein	115 (94.3)
Left femoral vein	7 (5.7)
Sedation during procedure	
Conscious sedation	101 (82.8)
General anesthesia	1 (0.8)
Sedation, type not identified	1 (0.8)
No sedation	19 (15.6)
Procedure duration, min	37.2 ± 17.7
Estimated blood loss, mL	
0-250	76 (62.3)
251-500	39 (32.0)
501-1000	5 (4.1)
1001-2000	2 (1.6)
Intraoperative transfusion	1 (0.8)
Thrombolytic use within 48 h of procedure^a^	3 (2.5)
ICU length of stay, d	1.5 ± 1.78
Hospital length of stay, d	5.3 ± 3.31

Values are mean ± SD or n (%).

a All thrombolytics were administered during the index procedure.

### Effectiveness end points

The mean 48-hour postprocedural RV/LV diameter ratio significantly decreased from 1.51 ± 0.31 at baseline to 1.07 ± 0.22 at 48 hours following use of the percutaneous mechanical aspiration thrombectomy device treatment. This equated to a −0.45 ± 0.27 (*P* < .001) decrease from baseline (Central Illustration). This improvement exceeded the primary prespecified goal of a −0.12 RV/LV diameter ratio change, and thus, the study achieved its primary effectiveness end point.

**Central Illustration:**
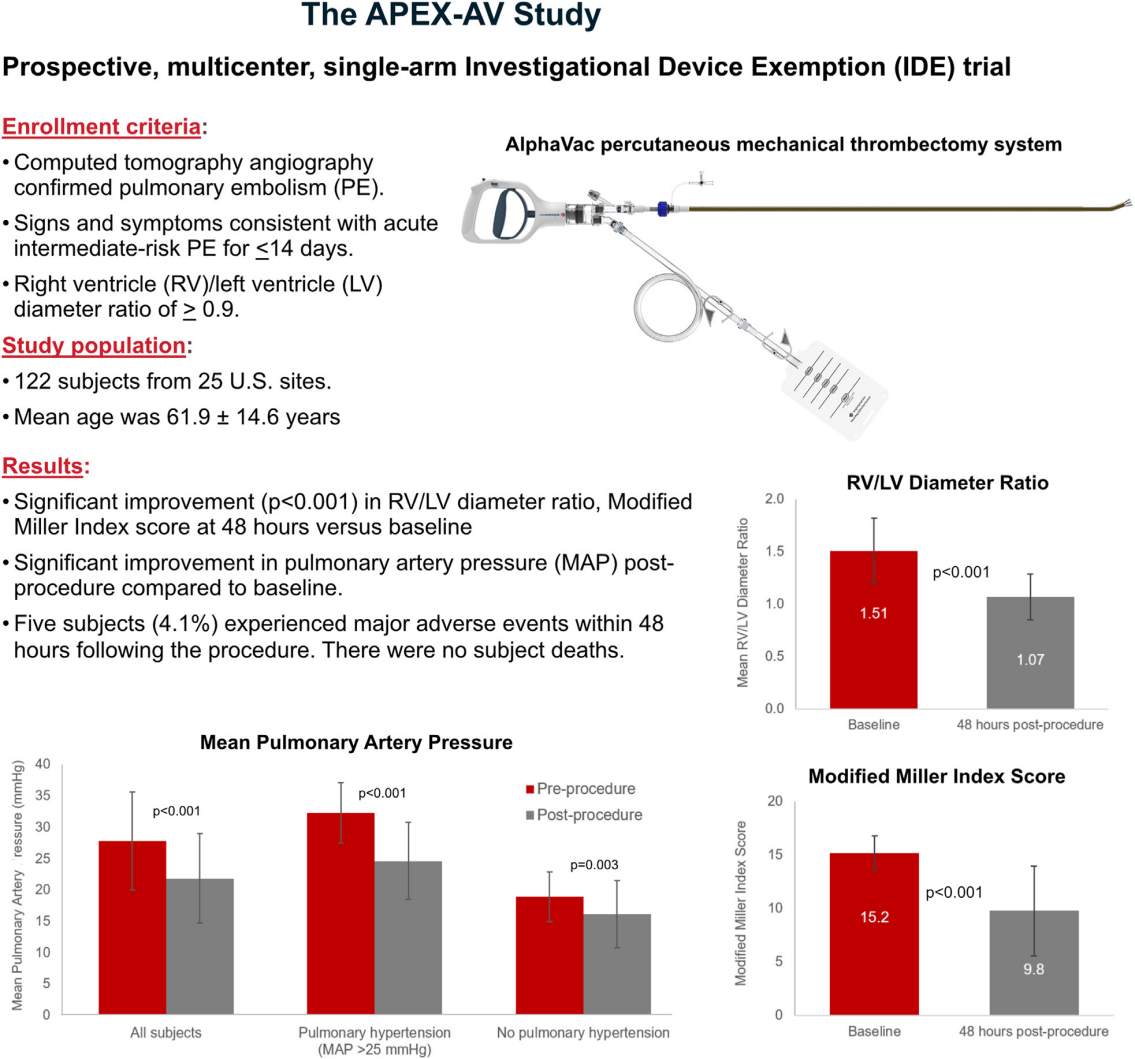
**TheAPEX-AVstudy.** APEX-AV, Acute Pulmonary Embolism extraction with the AlphaVac system.

**Figure 2:**
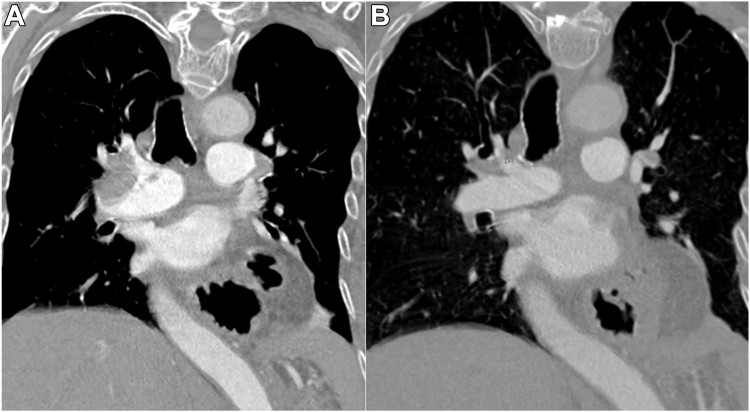
**Computed tomography scans illustrating a reduction in clot burden.** (A) Preprocedure and (B) postprocedure.

The mean modified Miller index score significantly decreased from 15.2 ± 1.6 at baseline to 9.8 ± 4.2 at 48 hours postprocedure (−5.6; *P* < .001). There was also a statistically significant decrease in the mean PAP from 27.8 ± 7.8 mm Hg before the procedure to 21.8 ± 7.2 mm Hg postprocedure (−6.1; *P* < .001). This improvement was more pronounced in subjects with pulmonary hypertension, defined as subjects with a mean PAP greater than 25 mg (−7.7; *P* < .001) compared with normotensive subjects (−2.8; *P* < 0.003) (Central Illustration). Figure 2 is an example of pre-CT and post-CT scans from a subject enrolled in the study, which illustrate a reduction clot burden.

### Safety end points

Five of the 122 subjects (4.1%) experienced a MAE as adjudicated by the clinical events committee during the 48-hour assessment period following the procedure. This 4.1% rate of subjects developing MAEs was statistically significantly lower than the rate of 25% (*P* < .001) defined for the primary safety end point. All subjects experiencing MAEs had major bleeding, which resolved within 1 day with 3 (2.5%) being considered possibly or definitely device related. One subject developed hypovolemic shock on study day 1 that resolved the next day. A subject also developed pulmonary hemorrhage due to a pulmonary vascular injury, which was probably device related and clinical deterioration due to respiratory failure. It was noted that the majority of MAEs occurred within the first 15 subjects treated. A post hoc analysis of the primary safety end point excluding these 15 subjects resulted in an MAE rate of 1.9% (2 subjects).

Overall, 18 (14.8%) subjects experienced a serious adverse event (SAE) during the study with 4 (3.3%) subjects developing device-related SAEs within 30 days of the procedure. Fifteen (12.3%) subjects experienced non–device-related SAEs. One subject experienced both a device-related SAE and a non–device-related SAE. One subject (0.8% of the 118 subjects where 30-day postprocedural follow-up data were available) developed symptomatic recurrence of their PE on day 4 following the procedure. There were no deaths and no unanticipated adverse device-related complications during the study.

Of the 4 subjects who developed a device-related SAE, 1 experienced significant bleeding at the catheter insertion site on study day 2 that resolved the next day. A second subject developed a low hemoglobin level that was considered nonserious of mild severity and possibly related to device. The subject’s hemoglobin was at 13.2 g/dL at screening and dropped to 8.8 g/dL postprocedurally following 400 mL of reported blood loss during the procedure. The subject’s hemoglobin dropped further to 6.9 g/dL but no bleeding or hematoma source was seen on the CTA and retroperitoneal bleeding was ruled out as per CT. The subject was transfused with 1 unit of red blood cells and their hemoglobin increased to 8.1 g/dL before discharge. The third subject developed hemoptysis in association with a pulmonary hemorrhage on study day 1, which the investigator believed was most likely caused by the large bore of the device and adjunctive anticoagulation. A follow-up CT scan at 48 hours showed no hemorrhage but the chest x-ray showed mild atelectasis. The patient also developed respiratory failure (clinical deterioration), which was considered to also probably be device related. A fourth subject experienced a device-related SAE characterized by a blood pressure drop during the procedure, which was likely due to back bleeding from the sheath that was in the main PA. The investigator indicated that this was partially user error because they did not realize the volume of blood leaking through the proprietary sheath. This resolved following a 1-L bolus of intravenous fluids and 2 units of packed red blood cells.

## Discussion

The APEX-AV trial reports the safety and efficacy of the new mechanical vacuum-assisted thrombectomy system, the AlphaVac F18^85^ system, for the treatment of patients with acute intermediate-risk PE. The observed −0.45 decrease in 48-hour RV/LV diameter ratio represented a 29.1% improvement from baseline, exceeding the predefined goal for this study based on previously reported results associated with the treatment of acute PE with anticoagulants.^4^, ^5^, ^6^, ^7^ This not only indicates an improvement in the RV function but also demonstrates comparable efficacy with other commercially available mechanical thrombectomy devices.^8^^,^^13^ Although a reduction in the RV/LV ratio has been shown to be a strong predictor of all-cause mortality and adverse outcomes. Further clinical trials are required to correlate this reduction with overall quality of life.^14^^,^^15^

The composite MAE rate of 4.1% exceeded the safety performance goal for the study. Most MAEs occurred within the first 15 subjects treated in the APEX trial with the rate of MAEs reducing to 1.9% thereafter. The study did not include any lead-in cases before the first patients being enrolled at each site. This demonstrates a short learning curve with this system. It is also noteworthy that 3 of the 5 major bleeding episodes were related to access site, which were believed to be related to the large bore of the device. Another important feature of this device is the ability to navigate the cannula from one PA to the other without a guide wire. While the study did not track the number of procedures performed without a guide wire, no complications were reported when the cannula was moved from one PA to another.

Vacuum-assisted thrombectomy (AngioVac system; AngioDynamics) has demonstrated the ability to safely and effectively remove right heart and intravascular thrombi and vegetations.^3^^,^^16^ The AlphaVac system is an off-the-shelf, easy to assemble, thrombectomy device that retains the benefits and features of the AngioVac system and eliminates the need for a perfusionist and additional support. The device is designed to enable immediate set up and shorter procedure times as seen in the lower device/procedure time (37.2 ± 17.7 minutes) reported in this study when compared with the 57.7 ± 29.6 minutes reported for the FLARE study, which used the FlowTriever Retrieval/Aspiration System (Inari Medical).^8^ The AlphaVac system has a handle that consists of a volume-limiting switch enabling users to select the aspiration volume (10 or 30 mL) per actuation. This limits blood loss as seen in this study with most patients (>90%) losing less than 500 mL of blood. The system also consists of an 18F cannula that has a funnel tip that expands to 33F and facilitates the ability to engage large thrombi and prevent clogging. The funnel tip not only captures the material but also, aided by the suction force applied through the cannula via the AlphaVac handle, compresses and “toothpastes” the material into the funnel, through the cannula, and ultimately into the waste bag. This is validated in this study with a 35.5% reduction in the clot burden as measured by the modified Miller index, which is approximately 3.5 times greater than the clot burden reduction reported in other mechanical thrombectomy IDE trials.^8^^,^^13^ Moreover, this higher reduction in clot burden was achieved without the routine utilization of tissue-plasminogen activator and was comparable with the clot burden reduction reported in trials using lytic-based therapies.^17^^,^^18^ This not only shortens ICU stay but also reduces the incidence of bleeding.^19^

**Figure 3:**
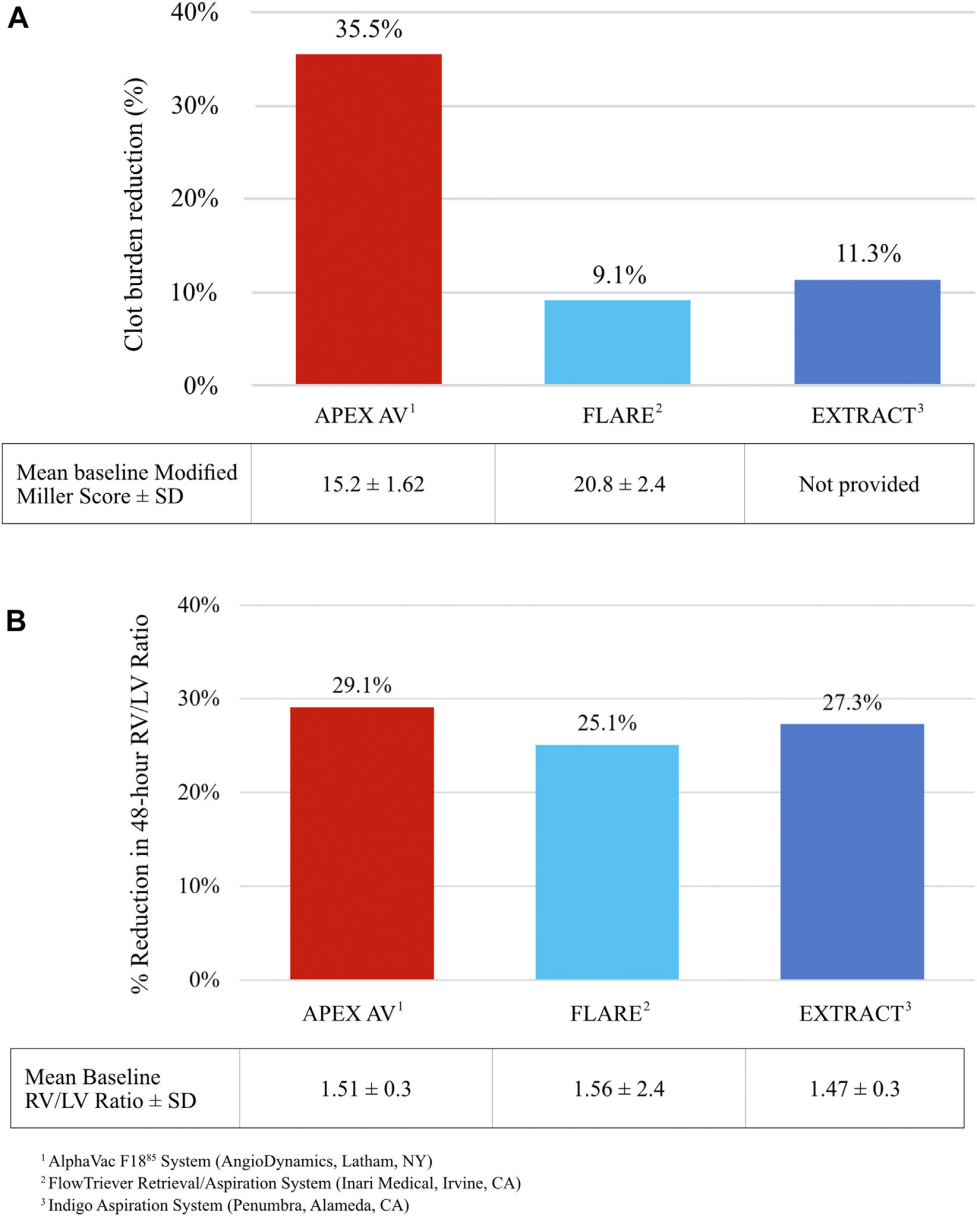
**Outcome comparisons between studies.** (A) Percent reduction in clot burden, and (B) Percent reduction in 48-hour right ventricle/left ventricle ratio.

Overall, the APEX trial demonstrated comparable safety and improvement in RV function with improved reduction in the clot burden and shorter procedural times when compared with other reported mechanical thrombectomy IDE trials (Figure 3).^8^^,^^13^ While larger, randomized controlled studies such as the PE-TRACT (NCT05591118), HI-PEITHO (NCT04790370),^20^ and PEERLESS (NCT05111613)^21^ trials are currently underway, there remains a glaring need for head-to-head trials to compare differences between available thrombectomy devices regarding safety and effectiveness and trial designs, which collect longer term data in order to correlate acute outcomes with patient quality of life.

### Study limitations

There are several limitations associated with this study. As a result of the lack of a control arm where conservative therapy with anticoagulation or alternative interventional approaches were used during the same time interval, the ability to compare the results observed in this study to other potential treatment strategies is limited. While subjects were prospectively enrolled, there was the potential for selection bias. The study also lacked long-term follow-up data beyond the 30-day observation period. Future studies in a broader population of high-risk patients with PE and direct comparison with alternative percutaneous devices used to treat PE would add to the understanding of the clinical utility for this technology.

## Conclusions

The results of this study confirm that performing vacuum-assisted aspiration thrombectomy with the AlphaVac system is a safe, efficient, and effective option for treating patients with acute intermediate-risk PE. This device provides an endovascular approach to treating this patient population, improving RV function and reducing clot burden score.
