# Supplementary material for: Novel Mechanical Aspiration Thrombectomy in Patients With Acute Pulmonary Embolism: Results From the Prospective APEX-AV Trial
Source: J Soc Cardiovasc Angiogr Interv. 2024 Dec 27;4(1):102463. doi: 10.1016/j.jscai.2024.102463 (PMC11887559; doi:10.1016/j.jscai.2024.102463)
Supplement: Supplemental Tables S1 and S2 [file mmc1.pdf]

Supplemental Table S1. APEX-AV Study Inclusion and Exclusion Criteria

Inclusion criteria:

- A signed and dated informed consent form.
- Subject is 18 years of age and older.
- Subject presents clinical signs and symptoms consistent with acute intermediate-risk pulmonary embolism for less than or equal to 14 days.
- Subject has a diagnosis of pulmonary embolism detected from computed tomography angiography (CTA).
- Subject has an RV/LV ratio of 0.9 or higher.
- Subject has a systolic blood pressure (SBP) of 90mmHg or higher
- Subject has a heart rate of 130 beats per minute (BPM) or less prior to the procedure.
- Subject is deemed medically eligible for interventional procedure(s) per institutional guidelines and/or clinical judgement.

Exclusion criteria:

1. Subjects who are or may be pregnant as determined by a positive pregnancy test or who are breastfeeding.
2. Subjects who have any contraindication to systemic or therapeutic doses of heparin
3. or anticoagulants.
4. Subjects who have used thrombolytics (tPA) in the past 30 days of baseline CTA.
5. Subjects who have pulmonary hypertension with peak pulmonary artery pressure (PAP) > 70 mmHg.
6. FiO2 requirement >40% or >6 LPM to keep oxygen saturations >90%
7. Subjects with hematocrit <28% within 6 hours of index procedure.
8. Subjects with platelets count < 100,000/µL.
9. Subjects with serum creatinine >1.8 mg/dL.
10. Subjects with International Normalized Ratio (INR) >3.
11. Subjects who have undergone a major trauma within the past 14 days of the index procedure and have Injury Severity Score (ISS) >15.
12. Subjects with the presence of cancer requiring active chemotherapy.
13. Subjects with known bleeding diathesis or coagulation disorder.
14. Subjects who have had cardiovascular or pulmonary surgery within the past 7 days of index procedure.
15. Subjects with a history of severe or chronic pulmonary hypertension, uncompensated heart failure, chest irradiation, underlying lung disease that is oxygen dependent, heparin-induced thrombocytopenia (HIT) and/or chronic left heart disease with left ventricular ejection fraction ≤ 30%.

16. Subjects with known anaphylactic reaction to radiographic contrast agents that cannot be pretreated.
17. Subject requires Vasopressor after fluids to keep pressure ≥ 90mmHg.
18. Subjects with left bundle branch block.
19. Subjects who have intracardiac lead in the right ventricle or atrium.
20. Evidence such as imaging or other that suggest the subject is not appropriate for this procedure.
21. Subjects that have life expectancy < 90 days.
22. Subjects dependent on extracorporeal life support such as extracorporeal membrane oxygenation (ECMO).
23. Participation in another investigational study

Supplemental Table S2. Definitions for Primary Safety Endpoints

Major Bleeding:

Bleeding was classified by the GUSTO15 (Global Utilization of Streptokinase and Tissue Plasminogen Activator for Occluded Coronary Arteries) bleeding criteria. Major bleeding included GUSTO Severe/life threatening and moderate categories:

- GUSTO Severe:
  - Intracranial hemorrhage
  - Fatal/life threatening bleeding
  - Resulting in substantial hemodynamic compromise requiring intervention
- GUSTO Moderate:
  - Requiring blood transfusion but did not result in hemodynamic compromise

Clinical Deterioration:

Also referred to hemodynamic collapse and defined as having one of the following:

- need for cardiopulmonary resuscitation, intubation, vasopressors, ECMO/ECLS; or systolic blood pressure (SBP)<90 mm Hg for at least 15 mins, or
- drop of SBP by at least 40 mm Hg for at least 15 minutes with signs of end-organ hypoperfusion (cold extremities or low urinary output<30 mL/h or altered mental status); or
- need for catecholamine administration to maintain adequate organ perfusion and SBP>90 mm Hg (including dopamine at the rate of >5 micrograms/kg per minute).

Pulmonary Vascular Injury:

Pulmonary vascular injury included the following events occurring in the pulmonary vasculature:

- Arterial Venous Fistula
- Dissection
- Hemorrhage (Intrapulmonary/ Intrapleural)
- Hemoptysis
- Intimal flap
- Perforation
- Rupture
- Thromboembolic occlusion resulting in permanent damage i.e., infarction

Cardiac Injury:

The following events were considered as possible cardiac injury:

- Acute heart failure
- Acute myocardial infarction
- Arrhythmia requiring intervention
- Cardiac hematoma
- Tricuspid or pulmonic valve damage
